# Small Posterior Cranial Fossa and Cerebellopontine Cistern Volumes Are Associated With Bilateral Trigeminal Neuralgia

**DOI:** 10.3389/fneur.2020.573239

**Published:** 2020-10-15

**Authors:** Jiayu Liu, Ruen Liu, Bo Liu, Jingru Zhou, Cungang Fan, Feng Jiao, Dongliang Wang, Fang Li, Bo Hei

**Affiliations:** ^1^Department of Neurosurgery, Peking University People's Hospital, Beijing, China; ^2^Department of Neurosurgery, Jiangxi provincial People's Hospital Affiliated to Nanchang University, Nanchang, China

**Keywords:** bilateral trigeminal neuralgia, posterior cranial fossa, cerebellopontine cisterns, microvascular decompression, neurovascular conflict

## Abstract

**Objective:** To investigate whether small volumes of the posterior cranial fossa and cerebellopontine cisterns are associated with bilateral trigeminal neuralgia (BTN) and to provide further knowledge regarding the etiology and treatment of this rare disease.

**Methods:** We retrospectively analyzed clinical data and imaging examination results for 30 BTN patients between January 2009 and December 2019. Thirty age- and sex-matched healthy individuals and 30 patients with unilateral trigeminal neuralgia (UTN) were selected as two control groups. The volume of the posterior cranial fossa (VPCF) and volumes of the cerebellopontine cisterns were measured using ITK-SNAP 3.0, which considers the cerebrospinal fluid (CSF) volume based on the region of interest (ROI). Preoperative and postoperative statuses were based on visual analog scale (VAS) pain scores and Barrow Neurological Institute (BNI) scores.

**Results:** A total of 30 patients (11 males; 19 females) were included, and the age of the BTN participants ranged from 41 to 77 (59.93 ± 9.89) years. The duration of TN ranged from 1 to 20 (5.36 ± 3.92) years, and the interval between the two sides ranged from 0 to 3 (1.10 ± 0.79) years. Three patients (10%) in the BTN group had familial trigeminal neuralgia, with no other hereditary history of neurological disorders. In BTN patients, with 25 (83.3%) cases on the left side and 26 (86.7%) on the right side, veins were identified in the operative field and regarded as the individual or offending vessel. The mean VPCF was significantly lower in the patients with BTN than in the healthy controls (4,813 ± 1,155 mm^3^ vs. 5,127 ± 1,129 mm^3^, *p* = 0.008). The volumes of the cerebellopontine cisterns on both sides were significantly smaller in the BTN patients than in the healthy controls (477 ± 115 mm^3^ vs. 515 ± 112 mm^3^ on the left side, *p* = 0.001; and 481 ± 114 mm^3^ vs. 515 ± 110 mm^3^ on the right side, *p* = 0.007). There was no significant difference between the BTN group and the UTN group in terms of the VPCF (4,843 ± 1,184 mm^3^ vs. 4,813 ± 1,155 mm^3^, *p* = 0.402), and there was also no significant difference between the two groups in terms of preoperative VAS pain scores or BNI scores.

**Conclusion:** Overcrowding in the posterior fossa will lead to closer neurovascular relations and, a higher incidence of NVC, and ultimately may be more likely to lead to TN. Veins are the common offending vessels that cause BTN; they might be associated with abnormal vascular development leading to NVC. Microsurgical vascular decompression (MVD) is a safe and effective method for the treatment of BTN, similar to UTN.

## Introduction

Trigeminal neuralgia (TN) is recurrent and intense pain in the region of the trigeminal nerve innervation (1). The pathogenesis of TN is believed to comprise neurovascular conflict (NVC) between the trigeminal nerve and adjacent blood vessels (2). By eliminating NVC, microsurgical vascular decompression (MVD) can successfully relieve pain. Several studies have shown that the small space of the posterior cranial fossa is associated with NVC, suggesting a correlation between overcrowding of the posterior cranial fossa and TN (3, 4).

In general, bilateral trigeminal neuralgia (BTN) is rare in clinical practice (5), though its occurrence has been reported as early as the 18th century (6). The pathogenesis remains controversial. Some scholars believe that the pathogenesis of this disease is different from that of UTN (7), but most consider that it is the same type of disease. Although one study showed that the mean MRI volumetry of the posterior cranial fossa was smaller in a BTN group than in a unilateral trigeminal neuralgia (UTN) (8), volume measurement does not fully consider individual differences. Indeed, this measurement is susceptible to the influence of head circumference and body shape. Moreover, as a limitation, the number of cases was small in that study. Cerebrospinal fluid volume (CSFV) is an effective space for the posterior cranial fossa. The CSFV in the posterior cranial fossa can be used to evaluate the effective space in this region, and can accurately reflect the degree of crowding. In this study, we measured and compared the degree of posterior cranial fossa crowding in Chinese patients with BTN and investigated whether small volumes of the posterior cranial fossa and cerebellopontine cisterns are associated with BTN. Additionally, clinical data for patients with BTN were analyzed to provide further knowledge regarding the etiology and treatment of this rare disease.

## Methods

### Patients

Clinical data for 30 patients with bilateral trigeminal neuralgia who received MVD were collected between January 2009 and December 2019 at the Department of Neurosurgery, Peking University People's Hospital, the Seventh Medical Center of PLA General Hospital and Characteristic Medical Center of Strategic Support Force. Patients with secondary or atypical TN and incomplete clinical data were excluded. Operations were carried out by the corresponding author Ruen Liu.

Age- and sex-matched healthy individuals and patients with UTN were selected as two control groups. Written informed consent was obtained from each participant, and the study was approved by the institutional review board of the hospitals.

### Magnetic Resonance Imaging

A preoperative MRI examination was performed in all cases, including 3D T1- and T2-weighted high-resolution sequences, for clear visualization of the trigeminal nerve and all vascular structures. The use of 3D time-of-flight magnetic resonance angiography (MRA) allowed the visualization of only vessels with high flow, which are principally arteries.

Imaging was conducted using a Discovery 750 3.0T (GE Healthcare, Waukesha, WI) MRI scanner. T1-weighted anatomical images in the sagittal plane were collected with a 3D fast spoiled gradient-echo sequence: repetition time (TR) = 4.9 ms, echo time (TE) = 2 ms, flip angle = 15°, field of view (FOV) = 240 mm, in-plane resolution = 1 * 1 mm^2^, slice thickness = 1 mm, and 170 slices. All scans were performed by the same imaging physician.

### Image Data Analysis

**Figure 1:**
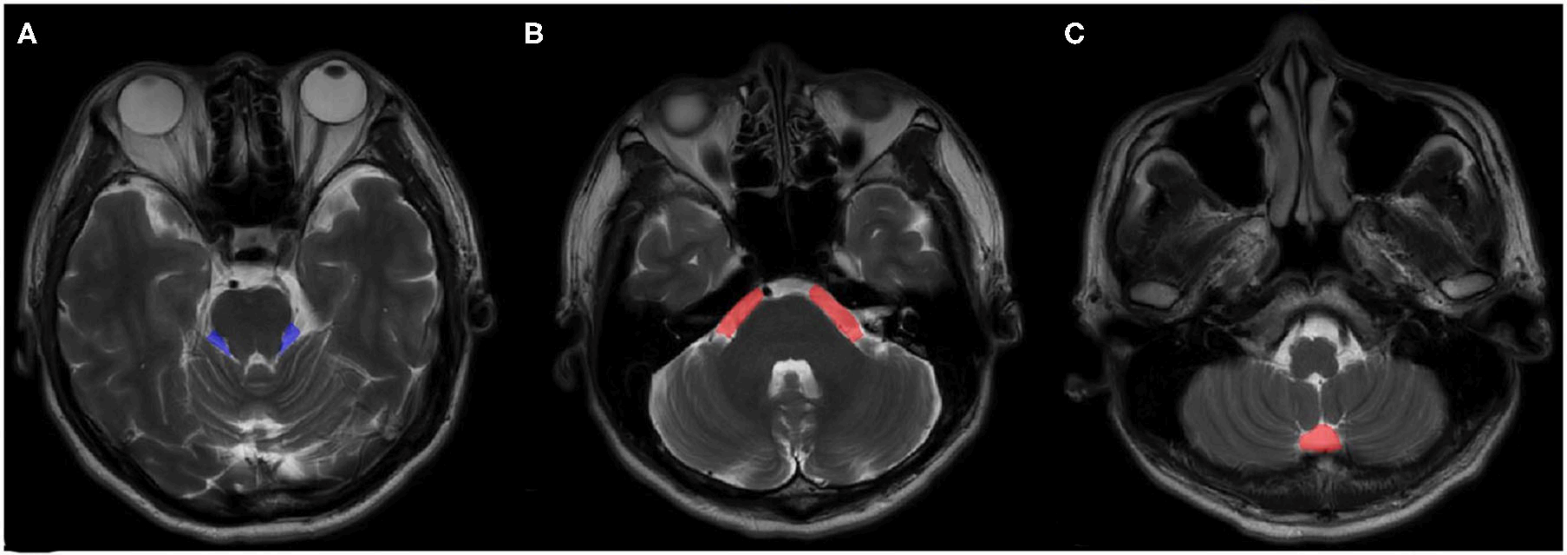
An example of ROI views on MRI. **(A)** cerebellopontine cisterns, **(B)** precerebellar cistern, **(C)** prespinal cistern.

VPCF was measured using ITK-SNAP 3.0 (Cognitica, Philadelphia, PA, USA, http://www.itksnap.org) that considered the cerebrospinal fluid (CSF) volume, as based on the region of interest (ROI), as well as the thickness and number of the individual layers. The measurement was limited to the region from the root entry zone (REZ) of the trigeminal nerve to that of the vagus nerve of the medulla oblongata and included only the fluid space of the precerebellar cistern, prespinal cistern and cerebellopontine cisterns (Figure 1). To minimize the influence of cerebellar atrophy, the CSF volume from behind the pontocerebellar cisterns, laterally, and from behind the cerebellar hemisphere as well as in the fourth ventricle were not considered for the determination of VPCF. The ROI was automatically marked on each layer in the 3D sequences and corrected manually by the radiologist, if necessary.

### Operative Technique

After the induction of general anesthesia, the patient was placed in the lateral park bench position with three-point fixation, and a retrosigmoid craniotomy was performed. The rostral edge of the craniotomy was extended until the caudal edge of the transverse sinus was visible and the junction between the transverse and posterior edges of the sigmoid sinus was adequately exposed. After opening the dura mater, the cerebellar horizontal fissure was carefully dissected to minimize retraction of the acoustic nerve. With maximum protection of the petrosal veins, the trigeminal nerve was observed, and we inserted a Teflon prosthesis between the offending vessels and the affected nerve to separate the neurovascular conflict. If the arcuate eminence of the petrosal bone was found to compress the trigeminal nerve during the operation, the abnormal bone was removed.

If no neurovascular conflict was observed during the operation, the patient underwent microvascular decompression with nerve combing. For nerve combing, the trigeminal nerve itself was then longitudinally divided along its fibers using a special nerve combing knife with a cutting edge of 0.90 mm into 4–5 bundles from the REZ to the petrous bone (9). For concomitant patients, we believe that the side with more severe symptoms should be treated first. Contralateral surgery should be performed if the contralateral side still has pain or aggravation after the first surgery (At least 3 months).

### Data Collection

Baseline and medical history data were obtained from medical records. The baseline data included age, sex, and preoperative and postoperative pain status according to visual analog scale (VAS) pain scores. VAS pain scores were recorded on a 11-point scale, with zero indicating no pain and 10 indicating maximal pain. Postoperative outcomes of TN were assessed by the Barrow Neurological Institute (BNI) pain intensity score and the BNI facial numbness score, and the total of both scores was considered for further analysis (10) (Table 1).

**Table 1:** Barrow Neurological Institute (BNI) pain intensity score, facial numbness score, and total evaluation of the results.

(P) Evaluation of pain relief by the BNI pain intensity score
1. No pain, no medication
2. Occasional pain, not requiring medication
3. Some pain, adequately controlled with medication
4. Some pain, not adequately controlled with medication
5. Severe pain/no pain relief
(N) Evaluation of numbness by the BNI facial numbness score
1. No facial numbness
2. Mild facial numbness, not bothersome
3. Facial numbness, somewhat bothersome
4. Facial numbness, very bothersome
(T) Total evaluation of results = (P) + (N)
2 Excellent
3 Good
4 Fair
≥5 Poor

### Statistical Analysis

SPSS statistical software 19.0 (IBM Corp., Armonk, NY, USA) was used for data analysis. Numerical variables are expressed as the mean ± SD. Qualitative variables are described as the absolute value of cases in the distinctive group. Statistical significance between quantitative variables was assessed by the χ^2^ test, with Yates's or Fisher's correction, if necessary. Student's *t*-test was performed to evaluate the data and to follow a normal distribution. Bonferroni correction was applied for multiple comparisons. Significant differences between groups were indicated at *p* < 0.05.

## Results

### Baseline Characteristics

A total of 30 patients (11 males; 19 females) were included, and the age of the BTN participants ranged from 41 to 77 (59.93 ± 9.89) years. Thirty age- and sex-matched healthy individuals and 30 patients with UTN were selected as two control groups. In the BTN group, 14 patients were initially affected on the left side and 16 on the right side. The duration of TN ranged from 1 to 20 (5.36 ± 3.92) years, and the interval between the two sides ranged from 0 to 3 (1.10 ± 0.79) years. Three patients (10%) in the BTN group had familial trigeminal neuralgia, but there was no other hereditary history of neurological disorders.

**Figure 2:**
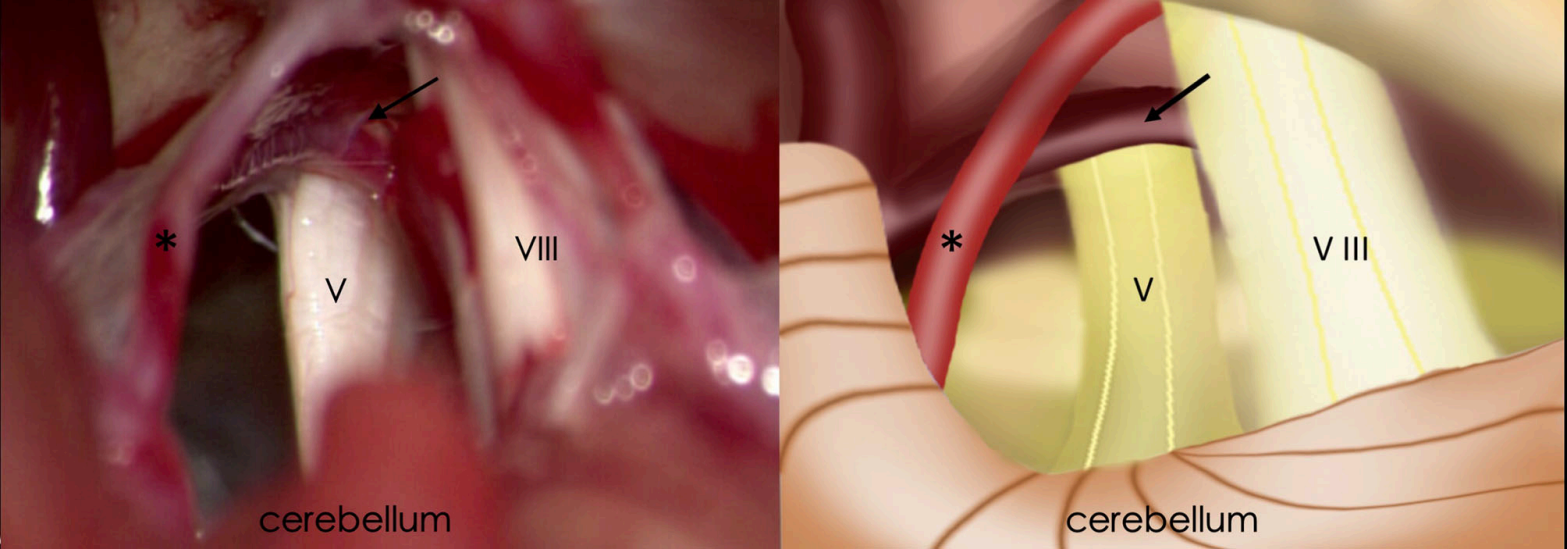
Intraoperative findings of retrosigmoid craniotomy **(Left)** and corresponding schematic diagram **(Right)**. Black arrow, the petrosal vein; Asterisk, superior cerebellar artery; V, trigeminal nerve; VIII, vestibulocochlear nerve.

Preoperative VAS scores were 9.17 ± 0.99 for the left side and 9.33 ± 0.76 for the right side. Among the BTN patients, 25 (83.3%) cases for the left side and 26 (86.7%) for the right side, veins were identified in the operative field and were regarded as the individual or offending vessel (Figure 2). In the other patients, the offending vessels were the anterior inferior cerebellar artery (AICA), superior cerebellar artery (SCA), SCA and AICA or none of these. The offending vessels in all three familial patients were veins. The follow-up period ranged from 6 to 110 months. For left-side cases, postoperative BNI scores were excellent (*T* = 2) in 25 patients (83%) and good (*T* = 3) in 5 (17%). For right-side cases, 24 (80%) patients had excellent outcomes, and 6 (20%) had good outcomes (Table 2). All the offending vessels in this study corresponded to TN on the same side. There is no case with bilateral neuralgia that improved bilaterally only with unilateral decompression.

**Table 2:** Clinical characteristics of the groups.

**Group**	**BTN (Left)**	**BTN (Right)**	**UTN**
VAS	9.17 ± 0.99	9.33 ± 0.76	9.23 ± 1.04
BNI			
2	25	24	26
3	5	6	4
Offending vessels			
SCA	2	1	19
AICA	1	0	3
SCA + AICA	0	1	3
Only Vein	18	19	3
Vein + SCA	4	3	0
Vein + AICA	2	2	0
Vein + SCA + AICA	1	2	0
None	2	2	2

No mortality, complete facial paralysis, intracranial haematoma, or postoperative hearing loss occurred, and there were no other serious complications. Two patients had scalp tingling, five had facial numbness, and one had transient vertigo, all of whom were cured by symptomatic and supportive treatment. In the follow-up period, no recurrence or any dysfunction of cranial nerves was found on either side. Furthermore, there was no significant difference between the two sides in terms of preoperative VAS pain scores (*p* = 0.316), offending vessels (*p* = 0.960) or BNI scores (*p* = 0.739).

### Volumes of the Posterior Cranial Fossa and Cerebellopontine Cisterns

VPCF was defined as the volumes of the precerebellar cistern, prespinal cistern and cerebellopontine cisterns on both sides in BTN patients. The mean VPCF was significantly lower in the patients with BTN than in the healthy controls (4,813 ± 1,155 mm^3^ vs. 5,127 ± 1,129 mm^3^, *p* = 0.008). Additionally, the volumes of the cerebellopontine cisterns on both sides were significantly smaller in the BTN patients than in the healthy controls (477 ± 115 mm^3^ vs. 515 ± 112 mm^3^ on the left side, *p* = 0.001; and 481 ± 114 mm^3^ vs. 515 ± 110 mm^3^ on the right side, *p* = 0.007). In contrast, there was no significant difference between the BTN and control groups in terms of the precerebellin cistern volume (3,084 ± 743 mm^3^ vs. 3,148 ± 721 mm^3^, *t* = −0.9, *p* = 0.370) or the prespinal cistern volume (771 ± 185 mm^3^ vs. 787 ± 180 mm^3^, *t* = −1.0, *p* = 0.400) (Table 3).

**Table 3:** Comparison of the volumes of the posterior cranial fossa and cerebellopontine cisterns between the BTN and healthy control groups.

**Group**	**BTN**	**Healthy controlled**	**Statistically**
			**significance**
Posterior fossa vol, mm^3^	4,813 ± 1,155	5,127 ± 1,129	*t* = −2.8; *p* = 0.008
CPA cistern vol, mm^3^			
Left	477 ± 115	515 ± 112	*t* = −3.5; *p* = 0.001
Right	481 ± 114	515 ± 110	*t* = −2.9; *p* = 0.007
Precerebellin cistern	3,084 ± 743	3,148 ± 721	*t* = −0.9; *p* = 0.370
vol, mm^3^			
Prespinal cistern	771 ± 185	787 ± 180	*t* = −1.0; *p* = 0.400
vol, mm^3^			

In the UTN group, the mean VPCF was significantly lower than that in the healthy control group (4,843 ± 1,184 mm^3^ vs. 5,127 ± 1,129 mm^3^, *t* = −2.61, *p* = 0.014). Moreover, the volumes of the cerebellopontine cisterns on the affected side were significantly smaller than those on the healthy side (472 ± 116 mm^3^ vs. 482 ± 121 mm^3^, *t* = −2.46, *p* = 0.020). However, there was no significant difference between the BTN group and the UTN group in terms of VPCF (*t* = −0.85, *p* = 0.402). There was also no significant difference between the BTN group and the UTN group in terms of the precerebellin cistern volume (3,084 ± 743 mm^3^ vs. 3,087 ± 759 mm^3^, *t* = −0.119, *p* = 0.906) or the prespinal cistern volume (771 ± 185 mm^3^ vs. 772 ± 190 mm^3^, *t* = −0.120, *p* = 0.900).

### Comparison Between the Bilateral TN and UTN Groups

For the 30 age- and sex-matched UTN patients, the preoperative VAS score was 9.23 ± 1.04. The superior cerebellar artery (SCA) was identified in the operative field in 26 (87%) patients, though veins were identified in only 3 (10%) patients. Postoperative BNI scores were excellent (*T* = 2) in 26 patients (87%) and good (*T* = 3) in 4 (13%) (Table 2). Conversely, there was no significant difference between the two groups in terms of preoperative VAS pain scores (*p* = 0.698 on the left side, *p* = 0.237 on the right side) or BNI scores (*p* = 0.718 on the left side, *p* = 0.488 on the right side).

## Discussion

Trigeminal neuralgia (TN), also known as painful convulsion, is mainly characterized by paroxysmal tearing and acute pain that occurs repeatedly in the trigeminal nerve distribution area on one side of the face (11), with an incidence of 8/100,000 (12). Currently, the theory of peripheral lesions has been accepted by most scholars (13). Regarding the pathogenesis of TN, it is believed that the trigeminal nerve becomes chronically compressed by abnormal twisted microvessels in the REZ area, resulting in inflammation and demyelination changes in the root of the trigeminal nerve and thus in a “short circuit” of membrane potential and neuropathic pain in the trigeminal nerve distribution area (14). One study (4) showed that the size, shape, and structure of the skull varies according to race and sex. Asians, especially women, have a higher incidence of TN and more crowding in the posterior fossa. The space occupied by tissues of the posterior fossa is small, resulting in NVC of the trigeminal nerve and clinical symptoms. However, for people with a large posterior fossa volume, there will be no symptoms of compression, clinical pain or other discomfort, even if the blood vessels are in contact with the nerve (15). In our study, the mean VPCF in the UTN group was significantly lower than that in the healthy control group (*p* < 0.05). The volumes of the cerebellopontine cisterns on the affected side were also significantly smaller than those on the healthy side (*p* < 0.05), similar to the results obtained by Rasche et al. (16) and Park et al. (17) based on two-dimensional measurement of the coronal plane and cross-section.

Bilateral TN is rare in clinical practice, and the proportion of BTN in TN patients is reported to be 0.6–5.3% (5). In the present study, patients with BTN often presented with paroxysmal needling, knife cutting or radiating excruciating pain on both sides, and the pain symptoms were similar to those of UTN. Furthermore, there was no significant difference between the two groups in terms of preoperative VAS pain scores (*p* > 0.05). BTN is often characterized by simultaneous or alternating attacks on both sides, and in our study, the interval between the two sides ranged from 0 to 3 years.

The pathogenesis of BTN is not clear, but several studies have suggested that BTN is significantly related to microvascular compression of the trigeminal nerve, which is consistent with unilateral pathogenesis (5–7). In this study, neurovascular conflict during surgery was recorded for 93% (28/30) of the patients (Table 2). The offending vessels observed were the AICA, SCA and veins. Some studies to date have linked BTN to multiple sclerosis (MS). For example, research by Brisman et al. reported the presence of MS in 4–10% of BTN patients (18), and Gale et al. found that ~8% of 210 patients with multiple sclerosis have trigeminal neuralgia (19). However, no patients in this study were found to have MS, which is consistent with the findings of another study of patients with BTN in the Chinese population (8). We also observed that the mean MRI volumetry of the posterior cranial fossa was smaller in the BTN group than in the UTN group, though with no significant difference between the groups for VPCF (*p* > 0.05). We believe that the reason for this conflicting outcome is that the measurement of volume did not fully consider individual differences. This measurement is susceptible to the influence of head circumference and body shape, and the number of cases was small, which is a limitation. As one study showed, the mean posterior fossa volume in ipsilateral TN patients was not different from that in controls, even though smaller cisterns were found in these patients (20). In contrast to our study, the method of measuring posterior fossae volume in the study by Park and Ha involved manual measurement by invasive image-guided surgery technology. In our study, the CSFV was used to evaluate the effective space of the posterior cranial fossa, accurately reflecting the degree of crowding. According to our results, the mean VPCF was significantly lower in the patients with BTN than in healthy controls, and the volumes of the cerebellopontine cisterns on both sides in BTN patients were significantly smaller (Table 3). Although previous studies have demonstrated that reduced posterior fossa volume is associated with UTN, our study is the first to find that small volumes of the posterior cranial fossa and cerebellopontine cisterns are associated with BTN. This finding strengthens the theory that BTN, similar to UTN, is the cause of neurovascular conflict. As mentioned above, trigeminal nerve compressed by vessels, resulting in inflammation and demyelination changes in the root of the trigeminal nerve and thus in a “short circuit” of membrane potential and neuropathic pain in the trigeminal nerve distribution area. Furthermore, 3 (10%) patients in the BTN group in our study had familial trigeminal neuralgia. Previous studies (5, 21) have shown that familial trigeminal neuralgia accounts for 7–17% of patients with BTN, which is consistent with the present results. In fact, anatomical abnormalities of the skull base may explain the familial incidence of TN (22). Takada et al. (23) reported 1 case of achondroplasia with TN, an autosomal inherited disease. The disease in this patient was due to skull dysplasia, which led to crowding of the posterior fossa and the production of NVC. Ugur et al. (24) described a case of Dandy Walker malformation with TN. Dandy Walker malformation is a congenital central nervous system malformation characterized by posterior fossa cysts and cerebellar vermis dysplasia, and it is considered a multifactorial genetic disease. This suggests that TN may have been caused by a small posterior fossa volume due to genetic factors in this patient. Although further study of familial BTN is needed, the results of this study suggest that a small posterior fossa volume is an important factor in the pathogenesis of BTN.

Although few patients have small pontine arteries or veins near the trigeminal nerve REZ that cause NVC, most cases of TN are caused by the SCA (25). UTN patients, 26 (87%) patients, the SCA was identified in the operative field in our study, which is consistent with the above finding. It is worth mentioning that in 25 (83.3%) left-side cases and 26 (86.7%) right-side cases, veins were identified in the operative field and were regarded as the individual or offending vessel. Moreover, the offending vessels in all three familial patients were veins. Smyth et al. (26) purported that familial TN is related to autosomal inherited vascular variant diseases. This study showed that the vascular variation of familial TN is different from known autosomal inherited vascular malformations, which might be related to variation of the vascular system in the posterior cranial fossa (26). Combined with the results of this study, BTN may also be associated with abnormal vascular development leading to NVC. However, further research is needed to address this possibility.

The significance of exploring the pathogenesis of BTN is to determine the surgical treatment strategy. Current studies suggest that MVD and radiofrequency thermocoagulation (RFT) are the most effective treatments (5–7). However, RFT of the bilateral trigeminal nerves inevitably results in a series of complications, particularly numbness in the bilateral trigeminal nerve distribution and decreases in masticatory function, which are often concerning to surgeons and patients. As NVC due to the small volume of the posterior fossa is the cause of BTN, we believe that MVD is the preferred effective treatment for patients who can tolerate craniotomy. Takada et al. (23) found that in patients with familial TN, MVD could achieve a better therapeutic effect in those with a small CPA caused by abnormal skull development. Additionally, MVD can preserve the function of the trigeminal nerve and reduce permanent dysfunction. Regardless, the disadvantage is the high recurrence rate. Combining the trigeminal nerve root in MVD is helpful to cure TN and reduce recurrence. In this study, microvascular decompression and nerve combing were used, and the clinical efficacy was satisfactory (9). Although some patients experienced mild facial numbness, they recovered within half a year. In addition, postoperative BNI scores were excellent (*T* = 2) in 25 patients (83%) with BTN on the left side and in 24 (80%) with BTN on the right side. Therefore, we believe that MVD is a safe and effective method for the treatment of BTN, similar to UTN. It is worth noting that as shown by our study, patients with BTN tend to have smaller cerebellopontine cisterns. The region of the cerebellopontine angle is the MVD operation area. When the posterior cranial fossa is crowded, the space decreases, and the operation becomes more difficult. Excessive traction of the cerebellar exposure field may damage nerves and blood vessels and increase postoperative complications. Venous compression is characterized by the fact that veins often adhere closely to nerves. Complete separation may cause blood vessel rupture, massive hemorrhage or, after cutting off blood vessels, cerebellar stem vein infarct hemorrhage; it may also injure nerves. Therefore, adhesion release should not be performed during the process, but removing the NVC is necessary. To better assess the difficulty of surgery and to help neurosurgeons develop the best treatment plan, it is recommended that the degree of posterior fossa crowding be evaluated before MVD.

In summary, overcrowding in the posterior fossa will lead to closer neurovascular relations and a higher incidence of NVC and ultimately may be more likely to lead to TN. The purpose of MVD is to separate the NVC by the surgical method and use Teflon for decompression. Therefore, we believe that MVD is an effective treatment for BTN compared with other treatment methods. However, the difficulty of surgery and postoperative complications will increase when the posterior fossa space, as the operating area of MVD, is crowded. Therefore, we recommend that the degree of posterior cranial fossa crowding should be evaluated before MVD to help the neurosurgeon determine the optimal surgical procedure. As described above, all patients in this study achieved satisfactory surgical results. Nonetheless, although this is the largest clinical study of this rare disease, BTN, to date, we must admit that the sample size was too small to enable comparison of the safety and availability of different treatments. In the future, multi-center large-sample studies or meta-analyzes are necessary to obtain effective conclusions.

## Conclusion

TN is a common and frequently occurring disease in neurosurgery, but BTN is rare in clinical practice. The etiology and treatment of BTN are still controversial. Although cases of bilateral trigeminal neuralgia have been reported, clinical studies on the etiology, diagnosis and treatment of BTN are currently lacking. Our study is the first to retrospectively analyze the clinical data, imaging examination results, surgical methods, and treatment efficacy for 30 Chinese patients. First, we found small volumes of the posterior cranial fossa and cerebellopontine cisterns to be associated with BTN. This finding strengthens the theory that BTN is the cause of neurovascular conflict. Second, veins were commonly the offending vessels that caused BTN, which might be associated with abnormal vascular development leading to NVC. Finally, our results show that MVD is a safe and effective method for the treatment of BTN.

## Data Availability Statement

The raw data supporting the conclusions of this article will be made available by the authors, without undue reservation.

## Ethics Statement

Written informed consent was obtained from the individual(s) for the publication of any potentially identifiable images or data included in this article.

## Author Contributions

JL and RL contributed to the writing of this manuscript, study conception and design, and the editing of the manuscript. BL, JZ, CF, FJ, DW, FL, and BH contributed to the editing of the manuscript. All authors contributed to the article and approved the submitted version.

## Conflict of Interest

The authors declare that the research was conducted in the absence of any commercial or financial relationships that could be construed as a potential conflict of interest.
